# Effect of admission hyperglycemia in sepsis patients with or without a history of diabetes

**DOI:** 10.1186/cc14451

**Published:** 2015-03-16

**Authors:** LA Van Vught, MA Wiewel, PM Klein Klouwenberg, AJ Hoogendijk, DS Ong, OL Cremer, MJ Bonten, MJ Schultz, T Van der Poll

**Affiliations:** 1 Academic Medical Center, Amsterdam, the Netherlands; 2 University Medical Center Utrecht, the Netherlands

## Introduction

Hyperglycemia is common and often multifactorial in critically ill patients. The association of hyperglycemia with adverse outcome has repeatedly been established in a variety of settings. The objective of this study was to investigate whether hyperglycemia on admission to the ICU impacts presentation and outcome of sepsis patients and whether this effect is different for patients with a history of diabetes mellitus.

## Methods

A two-center, prospective observational cohort study was conducted including all consecutive critically ill patients admitted to the ICU between January 2011 and July 2013. Sepsis patients were identified using strict clinical and diagnostic criteria. The first glucose measurement within a time window of 4 hours before up to 4 hours after ICU admission was categorized into euglycemia (71 to 140 mg/dl), mild hyperglycemia (141 to 200 mg/dl) or severe hyperglycemia (>200 mg/ dl), patients with hypoglycemia were excluded. A multivariable Cox proportional hazard model was used to determine the effect of admission hyperglycemia on mortality corrected for covariates.

## Results

Of the 1,059 patients admitted with sepsis, 526 (55.8%) had admission glucose levels within the normal range, 270 (25.5%) had mild hyperglycemia and 202 (19.1%) severe hyperglycemia. Patients with severe hyperglycemia were older, had higher APACHE IV scores and were more often diabetics compared with euglycemic patients. Shock on admission was more common in patients admitted with euglycemia. Crude mortality increased with increased admission glucose and a Cox regression analysis showed increased risk for 30day (HR = 1.67, CI = 1.24 to 2.23), 60-day (HR = 1.42, CI = 1.08 to 1.87) and 90-day (1.31, CI = 1.02 to 1.70) mortality in patients admitted with severe hyperglycemia compared with euglycemia. The association between mortality and severe hyperglycemia on admission was only present in patients without known diabetes but not in patients with a history of diabetes (30-day mortality HR = 1.67, CI = 1.15 to 2.43 vs. 1.84, CI = 0.97 to 3.49). Severe hyperglycemia was associated with a blunted proinflammatory cytokine response (IL-6 and IL-8) on admission in patients without, but not in patients with diabetes.

## Conclusion

Severe hyperglycemia on admission is associated with increased 30-day, 60-day and 90-day mortality in sepsis patients without a history of diabetes mellitus.

## Acknowledgement

This research was performed within the framework the Center for Translational Molecular Medicine (http://www.ctmm.nl), project MARS (grant 04I-201).

